# Clinical Outcomes of Discharged Patients With High Blood Pressure in the Emergency Department

**DOI:** 10.7759/cureus.76654

**Published:** 2024-12-30

**Authors:** Alhanouf Alsharif, Alanoud Aljohani, Sadeen Ashour, Ayman Zahim, Loui Alsulimani

**Affiliations:** 1 Medicine, King Abdulaziz University, Jeddah, SAU; 2 Emergency Medicine, King Abdulaziz University Hospital, Jeddah, SAU

**Keywords:** acute care, emergency department, high blood pressure, hypertension, patient outcomes

## Abstract

Background: Elevated blood pressure (BP) prompts immediate emergency department (ED) visits instead of outpatient care, thus constituting a high-weight concern for the ED. This study investigated the short- and long-term outcomes of high BP patients in the ED.

Methods: A retrospective cohort study was conducted at King Abdulaziz University Hospital (KAUH), reviewing ED visits from January to December 2022. A total of 156 patients with high BP were included, and data on demographics, visit disposition, and mortality were tracked 7, 30, 90, and 365 days after the visit.

Results: The mean patient age was 53.04±15.81 years; 78 (50%) were female and 80 (51.3%) were Saudi. Common complaints included headache (46, 29.5%), dizziness (21, 13.5%), and home-detected high BP (19, 12.2%). Notably, the majority (69, 44.2%) of the patients had BP >180/120 mmHg. Regarding those with return ED visits within 30 days, 48 (30.8%) were readmitted, one (2.1%) developed hypertensive encephalopathy, and four (8.3%) experienced renal failure.

Conclusion: High BP cases were a minor and often non-emergent reason for ED visits, with the majority being discharged home from the ED. These findings suggest the potential overutilization of EDs for non-emergent cases, highlighting the need for optimized resource allocation in hypertension management.

## Introduction

Hypertension (HTN), defined as a blood pressure (BP) of 140/90 mmHg or higher, is a global public health issue [1]. A systematic review and meta-analysis found that the prevalence of HTN in Saudi Arabia was 22.7%. This rate is significant but lower compared to other Middle Eastern countries, such as the United Arab Emirates (UAE; 24%), Oman (41.5%), Jordan (33.8%), and Lebanon (29.3%) [2].

According to guidelines from the American Heart Association and the American College of Cardiology, a significantly elevated BP of ≥180/120 mmHg, if coupled with evidence of target organ damage, is a hypertensive crisis. This can manifest in various forms, including hypertensive encephalopathy, intracerebral hemorrhage, acute ischemic stroke, acute myocardial infarction, acute left ventricular failure, unstable angina pectoris, dissecting aortic aneurysm, acute renal failure, or eclampsia. Conversely, hypertensive urgencies involve significantly elevated BP without organ function changes [3].

Elevated BP is a major concern that often prompts individuals to seek immediate medical attention at emergency departments (EDs) due to their continuous 24/7 availability [4-6]. The National Center for Health Statistics (NCHS) highlights that high BP cases are more frequently encountered in the ED than in outpatient primary care (43.5% vs. 27%) [7]. Annually, HTN-related ED visits reached a total of 27 million adult cases [8], with the majority not presenting as a true emergency. For example, a multicenter study revealed that only 25.3% of cases admitted to the ED for high BP were diagnosed as hypertensive emergencies [9]. These findings underscore the growing significance of ED visits due to HTN.

According to the 2023 Saudi Heart Association Guidelines, asymptomatic HTN does not require hospitalization. However, conditions such as acute aortic dissection, acute myocardial ischemia, or acute heart failure, indicative of hypertensive emergencies, necessitate hospital admission and immediate reduction of systolic blood pressure (SBP) through intravenous therapy. The treatment outcomes and factors linked with these cases have been a focus of research, underlining the complex relationship between HTN management and patient prognosis. A one-year follow-up study indicated that patients experiencing hypertensive crises had a higher incidence of cardiovascular morbidity and mortality, including coronary heart disease, stroke, and heart failure, compared to those with hypertensive urgencies [10]. This underscores the severe risk associated with organ damage in hypertensive crises. Specifically, mortality rates were 1% within 90 days and 2.5% within one year, with hospitalizations for potential hypertensive complications remaining below 1% for one year [11].

Extensive research on the short- and long-term outcomes of patients discharged from the ED with high BP in Saudi Arabia is lacking. Therefore, this study aimed to address the prevalence and the clinical outcomes of high BP in the ED of King Abdulaziz University Hospital (KAUH), Jeddah, Saudi Arabia, over a one-year period from January to December 2022.

## Materials and methods

Study design

This retrospective cohort study was conducted at KAUH in Jeddah, Saudi Arabia, in 2023. It included male and female patients who visited the ED with elevated BP, regardless of the complaint, from January to December 2022. Exclusion criteria were patients under the age of 18 years, those with incomplete medical records, and patients with duplicate files. HTN diagnoses were identified using the International Classification of Diseases, 10th Revision (ICD-10) codes I10, I11, I12, I13, I15, and R030. After applying these criteria to all ED visits, the study comprised 156 hypertensive patients. A comprehensive literature search was conducted using Google Scholar and PubMed with relevant keywords to identify prior related research. Ethical approval was granted by the Institutional Review Board (IRB) of KAUH in September 2023 (Reference No. 576-23).

Data collection

Patient data were initially extracted from the hospital’s information system (HIS) Phoenix (Phoenix Hospital Information Systems, Al-Anaiah International Company, Jeddah, Saudi Arabia), capturing both ED and inpatient data. Data collection was facilitated through Google Forms (Google LLC, Mountain View, CA, USA), a web-based survey tool. The collected information encompassed demographics (sex, age, and nationality), previous medical history (including HTN, diabetes mellitus, chronic obstructive pulmonary disease, heart failure, myocardial infarction, and stroke), antihypertensive medications (such as diuretics, calcium channel blockers, and angiotensin-converting enzyme inhibitors), and details of the ED visit (ambulance arrival, triage acuity, chief complaint, BP readings, and outcome). Additionally, the HIS was assessed for ED re-visits at intervals of seven, 30, 90, and 365 days looking for mortality, morbidity, and subsequent hospitalizations.

Data analysis

The data were analyzed using the Statistical Package for Social Sciences IBM SPSS Statistics for Windows, version 26.0 (IBM Corp., Armonk, NY, USA). To explore the associations between variables, the chi-squared test (χ2) was utilized for qualitative data, expressed as numbers and percentages. Quantitative data were presented as means and standard deviations (mean±SD), with the one-way analysis of variance (ANOVA) applied to parametric variables. A p-value of less than 0.05 was considered to indicate statistical significance.

## Results

During the study period, 156 patients with elevated BP presented in the ED. The characteristics of all patients during the study period are shown in Table 1. The mean age was 53.04±15.81 years, and half (78, 50%) were female. The majority of the patients (80, 51.3%) were of Saudi nationality. A significant proportion (124, 79.5%) of patients had previously been diagnosed with chronic diseases, of these, the majority (118, 95.1%) had HTN (Table 2). Over half of those patients (83, 53.2%) were on antihypertensive medications, among which calcium channel blockers were the most commonly used (51, 61.4%). Table 1 and Table 3 show patients’ arrival status and chief complaint: the vast majority of patients arrived at the ED using their own vehicle (155, 99.4%), while a single patient (0.6%) arrived by ambulance. Most of the patients were triaged with a Canadian Triage and Acuity Scale (CTAS) score of three (81, 51.9%). The most frequent chief complaints were headache 46 (29.5%), dizziness 21 (13.5%), and elevated BP measured at home (19, 12.2%). 

**Table 1:** Distribution of patient demographics and medical history (no. 156)

Variable	N (%)
Age (years) (mean±SD)	53.04±15.81
Sex	
Female	78 (50)
Male	78 (50)
Nationality	
Non-Saudi	76 (48.7)
Saudi	80 (51.3)
Previously diagnosed chronic diseases	
No	32 (20.5)
Yes	124 (79.5)
Arrival by ambulance	
No	155 (99.4)
Yes	1 (0.6)
Triage acuity	
High (1/2)	43 (27.6)
Intermediate (3)	81 (51.9)
Low (4/5)	32 (20.5)

**Table 2:** Distribution of chronic diseases and antihypertensive use HTN, hypertension

Those with chronic diseases (no. 124)	N (%)
HTN	118 (95.1%)
Diabetes mellitus	60 (48.3)
Chronic obstructive pulmonary disease	1 (0.8)
Heart failure	9 (7.2)
Stroke	9 (7.2)
Myocardial infarction	7 (5.6)
Antihypertensive use among all patients (no. 156)	
Yes	83 (53.2)
No	69 (44.2)
Couldn’t be determined	4 (2.6)
Those antihypertensive medications (no. 83)	
α₂-adrenergic agonist	5 (6)
Calcium channel blockers	51 (61.4)
Angiotensin receptor blocker	24 (28.9)
Angiotensin-converting enzyme inhibitor	34 (40.9)
Beta-blockers	26 (31.3)
Diuretics	26 (31.3)
Vasodilators	5 (6)

**Table 3:** Patients’ chief complaints during ED visits (no. 156) ED, emergency department

Chief complaint	N (%)
Headache	46 (29.5)
High BP measured at home	19 (12.2)
Abdominal pain	7 (4.5)
Back pain	2 (1.3)
Cervical muscle strain	1 (0.6)
Chest pain	11 (7.1)
Conjunctival hemorrhage	1 (0.6)
Constipation	1 (0.6)
Dizziness	21 (13.5)
Dyspepsia	1 (0.6)
Fever	1 (0.6)
Malaise and fatigue	9 (5.8)
Nausea and vomiting	2 (1.3)
Edema	1 (0.6)
Palpitations	1 (0.6)
Prescription refill	1 (0.6)
Seizure	1 (0.6)
Shortness of breath	25 (16)
Slurred speech	2 (1.3)
Visual disturbances	3 (1.9)

The data showed that 44.2% of patients experienced significantly elevated BP of ≥180/120 mmHg, while 42.3% presented with stage 2 HTN (BP >140/90 mmHg). In Table 4, 119 (76.3%) of patients were treated and discharged from the ED, and 33 (21.2%) were admitted due to HTN complications or other conditions. Among the admitted patients (n=33), heart failure was the most frequent final diagnosis upon admission, accounting for eight (24.2%) cases. Nearly one-third of the patients (48, 30.8%) were readmitted after their initial discharge, with 17 (33.5%) of these readmissions occurring within 30 days (Table 5). The main cause for these readmissions was elevated BP (19, 39.5%), and only one patient was diagnosed with hypertensive encephalopathy (2.1%).

**Table 4:** Patients’ current ED visit outcomes (no. 156) ED, emergency department

Hospital outcome	N (%)
Admitted to hospital	33 (21.2)
Death	1 (0.6)
Discharge against medical advice	2 (1.3)
Discharged from the ED	119 (76.3)
Left before treatment	1 (0.6)
Final diagnosis in hospitalization admission (no. 33)	
Acute myocardial infarction	1 (3)
Conn's disease	1 (3)
Diabetic ketoacidosis	1 (3)
Heart failure	8 (24.2)
Hypertensive emergency-induced pulmonary edema	2 (6)
Hypertensive encephalopathy	4 (12)
Nephrolithiasis	1 (3)
Meningioma	1 (3)
Renal failure	7 (21)
Chronic congestive splenomegaly	1 (3)
Respiratory failure	2 (6)
Risk for acute chest syndrome	1 (3)
Stroke	3 (9)

**Table 5:** Patients’ re-visit and diagnosis after ED discharge (no. 156) BP, blood pressure

Re-visit the ED	N (%)
No	108 (69.2)
Yes	48 (30.8)
Period of the re-visit: (no. 48)	
7 days	10 (20.8)
30 days	17 (33.5)
90 days	14 (29.1)
365 days	7 (16.6)
Re-visit final diagnosis (no. 48)	
Atrial fibrillation	1 (2.1)
Conn's disease	1 (2.1)
Elevated BP	19 (39.6)
Hypertensive encephalopathy	1 (2.1)
Obstructive sleep apnea	1 (2.1)
Renal failure	4 (8.3)
Syncope and collapse	1 (2.1)
Other (unrelated)	20 (41.7)

A higher percentage of patients with no chronic diseases had a BP ≥180/120 mmHg during their initial visit (p=0.001; Table 6). These patients were also found to have a higher likelihood of CTAS 3, experiencing chest pain, and being on antihypertensive medication (p-values <0.001, 0.012, and <0.001, respectively; Table 7 and Table 8). Additionally, they had a significantly higher prevalence of heart failure as a final diagnosis upon hospital admission (p=0.039; Table 9). Furthermore, patients with stage 1 HTN were significantly more likely to have diabetes mellitus (p=0.002; Table 6).

**Table 6:** Relationship between BP status and patient demographics (no. 156) *One-way ANOVA test HTN, hypertension; BP, blood pressure

Variable	ED visit elevated BP				χ2	p-value
	Elevated	Stage 1	Stage 2	Significantly elevated (≥180/120 mmHg)		
Age (years) (mean±SD)	59.77±16.83	60.88±16.24	50.61±15.42	53.19±15.61	2*	0.116
Sex						
Female	4 (30.8)	4 (50)	34 (51.5)	36 (52.2)	2.11	0.549
Male	9 (69.2)	4 (50)	32 (48.5)	33 (47.8)		
Nationality						
Non-Saudi	9 (69.2)	6 (75)	26 (39.4)	35 (50.7)	6.8	0.078
Saudi	4 (30.8)	2 (25)	40 (60.6)	34 (49.3)		
Previously diagnosed chronic diseases						
No	10 (76.9)	8 (100)	43 (65.2)	63 (91.3)	16.34	0.001
Yes	3 (23.1)	0 (0.0)	23 (34.8)	6 (8.7)		
Those with chronic disease (no. 124)						
HTN	10 (100)	8 (100)	39 (90.7)	61 (95.3)	2.28	0.516
Diabetes mellitus	6 (46.2)	6 (75)	18 (27.3)	30 (43.5)	20.34	0.002
Chronic obstructive pulmonary disease	0 (0.0)	0 (0.0)	1 (2.3)	0 (0.0)	1.89	0.594
Heart failure	2 (20)	1 (12.5)	1 (2.3)	5 (7.9)	4.33	0.227
Stroke	1 (10)	1 (12.5)	3 (7)	4 (6.3)	0.52	0.914
Myocardial infarction	1 (10)	1 (12.5)	1 (2.3)	4 (6.3)	2.01	0.57
Arrival by ambulance						
No	13 (100)	8 (100)	66 (100)	68 (98.6)	1.26	0.737
Yes	0 (0.0)	0 (0.0)	0 (0.0)	1 (1.4)		
Triage acuity						
High (1/2)	3 (23.1)	3 (37.5)	7 (10.6)	30 (43.5)	35.27	<0.001
Intermediate (3)	4 (30.8)	3 (37.5)	37 (56.1)	37 (53.6)		
Low (4/5)	6 (46.2)	2 (25)	22 (33.3)	2 (2.6)		

**Table 7:** Relationship between BP status and chief complaints (no. 156) HTN, hypertension; BP, blood pressure

Variable	ED visit elevated BP				χ2	p-value
	Elevated	Stage 1	Stage 2	Significantly elevated (≥180/120 mmHg)		
Chief complaint						
Abdominal pain	0 (0.0)	1 (12.5)	3 (4.5)	3 (4.3)	13.75	0.012
Back pain	1 (7.7)	1 (12.5)	0 (0.0)	0 (0.0)		
Cervical muscle strain	0 (0.0)	0 (0.0)	0 (0.0)	1 (1.4)		
Chest pain	1 (7.7)	0 (0.0)	4 (6.1)	6 (8.7)		
Conjunctival hemorrhage	0 (0.0)	0 (0.0)	1 (1.5)	0 (0.0)		
Constipation	0 (0.0)	0 (0.0)	0 (0.0)	1 (1.4)		
Dizziness	1 (7.7)	0 (0.0)	10 (15.2)	10 (14.5)		
Dyspepsia	0 (0.0)	0 (0.0)	0 (0.0)	1 (1.4)		
Fever	0 (0.0)	0 (0.0)	1 (1.5)	0 (0.0)		
Headache	2 (15.4)	0 (0.0)	27 (40.9)	17 (24.6)		
HTN	0 (0.0)	0 (0.0)	7 (10.6)	12 (17.4)		
Malaise and fatigue	4 (30.8)	2 (25)	2 (3)	1 (1.4)		
Nausea and vomiting	0 (0.0)	0 (0.0)	1 (1.5)	1 (1.4)		
Edema	0 (0.0)	0 (0.0)	0 (0.0)	1 (1.4)		
Palpitations	0 (0.0)	0 (0.0)	1 (1.5)	0 (0.0)		
Prescription refill	1 (7.7)	0 (0.0)	0 (0.0)	0 (0.0)		
Seizure	0 (0.0)	0 (0.0)	1 (1.5)	0 (0.0)		
Shortness of breath	3 (23.1)	4 (50)	7 (10.6)	11 (15.9)		
Slurred speech	0 (0.0)	0 (0.0)	0 (0.0)	2 (2.9)		
Visual disturbances	0 (0.0)	0 (0.0)	1 (1.5)	2 (2.9)		

**Table 8:** Relationship between BP status and medical history (no. 156) BP, blood pressure

Variable	ED visit elevated BP				χ2	p-value
	Elevated	Stage 1	Stage 2	Significantly elevated (≥180/120 mmHg)		
Antihypertensive use						
Yes	6 (46.2)	5 (62.5)	29 (43.9)	43 (62.3)	31.08	<0.001
Antihypertensive medications (no. 83)						
α₂-adrenergic agonist	1 (16.7)	0 (0.0)	1 (3.4)	3 (7)	1.93	0.587
Calcium channel blockers	1 (16.7)	2 (40)	18 (62.1)	30 (69.8)	7.31	0.063
Angiotensin receptor blocker	1 (16.7)	2 (40)	5 (17.2)	16 (37.2)	4.09	0.251
Angiotensin-converting enzyme inhibitor	2 (33.3)	3 (60)	10 (34.5)	19 (44.2)	1.58	0.663
Beta-blockers	2 (33.3)	0 (0.0)	6 (20.7)	18 (41.9)	6.03	0.11
Diuretics	3 (50)	2 (40)	4 (13.8)	17 (39.5)	6.63	0.084
Vasodilators	0 (0.0)	0 (0.0)	0 (0.0)	5 (11.6)	4.94	0.176

**Table 9:** Relationship between BP status and hospital outcomes (no. 156) BP, blood pressure

Variable	ED visit elevated BP				χ2	p-value
	Elevated	Stage 1	Stage 2	Significantly elevated (≥180/120 mmHg)		
Hospital outcome						
Admitted to hospital	3 (23.1)	2 (25)	5 (7.6)	23 (33.3)	19.43	0.079
Death	0 (0.0)	0 (0.0)	0 (0.0)	1 (1.4)		
Discharge against medical advice	0 (0.0)	0 (0.0)	0 (0.0)	2 (2.9)		
Discharged from the ED	10 (76.9)	6 (75)	60 (90.9)	43 (62.3)		
Left before treatment	0 (0.0)	0 (0.0)	1 (1.5)	0 (0.0)		
Final diagnosis in hospitalization admission (no. 33)						
Acute myocardial infarction	0 (0.0)	0 (0.0)	0 (0.0)	1 (1.4)	15.88	0.039
Conn's disease	0 (0.0)	0 (0.0)	0 (0.0)	1 (1.4)		
Diabetic ketoacidosis	0 (0.0)	0 (0.0)	1 (1.5)	0 (0.0)		
Heart failure	2 (15.4)	1 (22.5)	1 (1.5)	4 (5.8)		
Hypertensive emergency-induced pulmonary edema	0 (0.0)	0 (0.0)	0 (0.0)	2 (2.9)		
Hypertensive encephalopathy	0 (0.0)	0 (0.0)	0 (0.0)	4 (5.8)		
Kidney stones	0 (0.0)	0 (0.0)	0 (0.0)	1 (1.4)		
Meningioma	0 (0.0)	0 (0.0)	0 (0.0)	1 (1.4)		
Renal failure	0 (0.0)	0 (0.0)	1 (1.5)	6 (8.7)		
Chronic congestive splenomegaly	0 (0.0)	1 (22.5)	0 (0.0)	0 (0.0)		
Respiratory failure	1 (7.7)	0 (0.0)	1 (1.5)	0 (0.0)		
Risk for acute chest syndrome	0 (0.0)	0 (0.0)	1 (1.5)	0 (0.0)		
Stroke	0 (0.0)	0 (0.0)	0 (0.0)	3 (4.3)		

There was no significant relationship between the duration of ED readmission within seven, 30, 90, and 365 days and the admission diagnosis overall (p=0.056). Similarly, although a higher percentage of patients readmitted after discharge presented with elevated BP at home, the relationship between the duration of ED readmission and having elevated BP was not significant (p=0.238).

## Discussion

This study’s exploration of the clinical outcomes of patients presenting with high BP underscores the complexity and variability in outcomes and management strategies, highlighting a significant public health concern. Our findings that 76.3% of patients were discharged home, with a majority experiencing no subsequent severe complications, align with Frei et al.’s observations, which suggest a low risk of serious outcomes within seven days of readmission for hypertensive patients without serious complaints [12]. This implies an effective triage process that discerns between patients needing immediate hospitalization and those manageable via outpatient care, possibly indicating an overutilization of ED resources for conditions that do not require emergency intervention.

Vadeboncoeur et al. also highlighted that many patients self-refer to the ED for hypertensive concerns, similar to our finding that most of our study population initially presented to the ED with headaches rather than elevated BP at home [13]. This underscores the importance of educating patients on proper BP management and determining when ED consultation is warranted. Moreover, Ayalon-Dangur et al.’s findings, which show a link between elevated BP visits to the ED and subsequent heart failure, emphasize the necessity of effective management for these patients [14]. This is particularly relevant in our study, where a significant portion of patients presented with a BP ≥180/120 mmHg, potentially increasing their risk for short- and long-term adverse outcomes.

The high readmission rate within 30 days after the initial ED visit notably underscores the need for follow-up. Follow-ups through outpatient departments, healthcare centers, or the telephone within two to four weeks of discharge are crucial, as regular follow-up significantly correlates with controlled BP among hypertensive patients [15]. Additionally, the correlation between initially elevated BP and a diagnosis of heart failure upon hospital admission highlights the complexities of HTN management. These findings advocate for a multidisciplinary approach to HTN care outside the ED, emphasizing timely interventions, patient education, and rigorous follow-up strategies to prevent the progression of HTN to more severe cardiovascular conditions.

This study has several limitations that could impact the interpretation and generalizability of its findings. First, its small population size limits the statistical power and ability to detect smaller effect sizes within the data. The modest number of patients included in our analysis may not fully represent the broader population of individuals with elevated BP visiting the ED, which restricts our capacity to generalize findings to all hypertensive patients seeking emergency care. Second, BP measurements were taken from hospital records, which might not accurately reflect the dynamic nature of BP changes over time. This data collection method may overlook the variations in BP that could influence clinical decision-making and patient outcomes.

## Conclusions

In conclusion, HTN remains one of the most common diseases worldwide, prompting many patients to visit the ED when experiencing elevated BP. Most of the patients will be discharged safely from the ED, but concerns persist about readmitting patients due to HTN and its complications, which highlight the complexities associated with the discharge process and its potential link to increased mortality and morbidity. These risks include potential readmissions due to heart failure, renal failure, or encephalopathy, alongside variability in the duration until readmission, emphasizing the challenges of managing discharged hypertensive patients. Future research should expand the scope of the investigation to include a larger patient cohort across multiple hospitals and emphasize direct patient interviews for more accurate and informative data collection.
